# Supplementary material for: Resolving the full spectrum of human genome variation using Linked-Reads
Source: Genome Res. 2019 Apr;29(4):635–45. doi: 10.1101/gr.234443.118 (PMC6442396; doi:10.1101/gr.234443.118)
Supplement: Supplemental Material [file supp_gr.234443.118_Supplemental_longranger-2.2.zip › longranger-2.2.tar.bz2!/lr2.2-public/lib/stan/cmdstan/stan/doc/api/html/namespaces.html]

Stan: Namespace List


|  |  |
| --- | --- |
|  | Stan  2.5.0  probability, sampling & optimization |


- Main Page
- Related Pages
- Namespaces
- Classes
- Files

- Namespace List
- Namespace Members


All Classes Namespaces Files Functions Variables Typedefs Enumerations Enumerator Friends Macros Pages

Namespace List

Here is a list of all namespaces with brief descriptions:

[detail level 1234]

|  |  |
| --- | --- |
| boost | Reimplementing boost functionality |
| math | Reimplmeneting boost functionality for `stan::agrad::var` and and bugs in classification of integer types |
| Eigen | (Expert) Numerical traits for algorithmic differentiation variables |
| internal | (Expert) Product traits for algorithmic differentiation variables |
| stan | Probability, optimization and sampling library |
| agrad | Function gradients via reverse-mode automatic differentiation |
| common |  |
| recorder |  |
| gm | Directed graphical model compiler |
| io | Input/output utilities |
| json |  |
| math | Matrices and templated mathematical functions |
| detail |  |
| policies | Extending boost functionality |
| detail | Reimplementing boost::math::policies::detail::raise\_domain\_error |
| mcmc | Markov chain Monte Carlo samplers |
| memory | (Expert) Allocate, free, and manage underlying system memory |
| model | For compiling models |
| optimization |  |
| prob | Templated probability distributions |
| std | Template specification of functions in std for Stan |


---

[ Stan Home Page ]

*© 2011–2014,
Stan Development Team.*
